# Investigation of the Dynamics of Cavitation Bubbles in a Microfluidic Channel with Actuations

**DOI:** 10.3390/mi13020203

**Published:** 2022-01-28

**Authors:** Xiaopeng Shang, Xiaoyang Huang

**Affiliations:** School of Mechanical and Aerospace Engineering, Nanyang Technological University, Singapore 639798, Singapore; mxhuang@ntu.edu.sg

**Keywords:** bubble dynamics, coalescence, breakup, cavitation bubble, microfluidics

## Abstract

This work presents experimental and numerical studies on the dynamics of cavitation bubbles in a nozzle-shaped microfluidic channel with PZT (lead-zirconate-titanate) actuations. It is found that a cloud of bubbles can be generated near the center of the microfluidic channel when the actuation voltage is larger than a threshold at 1 kHz. After being generated, the bubbles under actuations oscillate radially with violent expansion and compression, and simultaneously translate upstream towards the opening of the nozzle. Along with radial oscillation and translation, the bubbles undergo frequent and drastic coalescence and breakup, leading to vigorous churning of surrounding liquids. The pressure variation and distribution in the microchannel are calculated by numerical simulation in Ansys Fluent, and results show that there is a low-pressure zone inside the microfluidic channel within each cycle of the actuation period, which is responsible for bubble generation observed in the experiments. The method of bubble generation in this study is novel and can be applied for the enhancement of heat and mass transfer in microfluidic operations.

## 1. Introduction

Microfluidics, which is involved in the science and technology of manipulating small amounts of fluids in networks of micro channels, has been broadly applied in different fields, such as chemistry, medicine and biology, owing to its simplicity, high sensitivity, low cost, and low sample consumption, etc. [1]. Heat and mass transfer exist widely in microfluidic operations. Due to small geometric dimensions, extremely low flow rates, and dominance of viscosity over inertia, the Reynolds number (Re) in microfluidics is usually very low, leading to laminar or even creeping flows. Thus, finding how to enhance the heat and mass transfer in microfluidic flows, so as to improve sample mixing, biochemical reaction and flow control, etc., remains an attractive topic.

A number of studies aiming at enhancing heat and mass transfer in microfluidics have been reported, and the developed methods are typically divided into two categories, passive and active methods. In the former, enhancement of heat and mass transfer relies on the advection flow caused by complex configurations or geometries of microchannels, such as herringbone structures, zigzag turns, serpentine channels and flow focusing systems and so on [2,3]. In spite of simple operation and low costs, a passive method requires a complex geometry design and very high flow rates to produce flow disturbances in a microchannel. If the requirements for the flow rate or working fluids are changed, a new design might be needed. In the latter category, an external actuation, such as magnetic forcing, electrokinetic forcing, an acoustic field, and even the thermal effect, is introduced to perturb the microfluidic flows to enhance the heat and mass transfer [4,5,6]. Park and Wereley [7] developed an opto-electric vortex generator with two coplanar electrodes. When a laser spot is focused on any region of exposed electrodes, a complex, three-dimensional vortex flow is formed rapidly. This technique is promising to produce desirable fluid flows and improve chemical and biological reactions through enhancing mass transfer in microchannels. Ahmed et al. [8] designed an acoustic micro-mixer by introducing an external air bubble within a “horse-shoe” structure of a microfluidic channel. Acoustic waves induce vibration of the trapped air bubble at its resonance frequency and cause a bulk flow around the air–liquid interface. Thermal disturbance can also act as the driving force to enhance the heat and mass transfer in microfluidic channels. Kim et al. [9] created multi-vortices by using the natural convection in conjunction with alternating heating, and the resultant vortical flow is successfully used for the mixing of samples for polymerase chain reaction (PCR) tests. Active methods have fewer limits on flow conditions and higher efficiency compared to passive methods. However, special equipment and elaborate manipulation are usually indispensable, which causes inconvenience in real applications. Another downside is that some active methods are primarily for fluids or particles with specific properties. For example, the electrokinetic and magnetic methods have requirements regarding the conductivity and magnetism of working fluids, respectively. The thermal method may damage the reagents and samples which are sensitive to the temperature variation; acoustic methods need to introduce and trap external gas in advance and there is also a problem of temperature rise at high acoustic frequency. Therefore, despite so many types of passive and active methods, development of novel and simple methods for the enhancement of heat and mass transfer in microfluidic systems remains to be an attractive research topic.

Bubble dynamics has been broadly applied in lots of microfluidic processes, such as micropumping, micromixing, flow control and manipulation of micron-sized objects [10,11,12]. Air bubbles of micron sizes are introduced into a microfluidic channel integrated with PZT (lead-zirconate-titanate) transducers. The surface of an air bubble exposed to PZT actuations functions as a vibrating membrane, causing a bulk movement in liquid near the air–liquid interface. This effect is known as cavitation microstreaming [13]. Rasouli and Tabrizian [14] reported an ultra-fast micromixer for the synthesis of organic nanoparticles based on cavitation microstreaming of oscillatory bubbles. The straightforward fabrication of the device coupled with efficient energy consumption, high controllability, and rapid mixing renders this mixer a practical platform for a myriad of nano and biotechnological applications. Guan and Sun [15] demonstrated a microfluidic mixing platform for low- and high-viscosity fluids by integrating oscillating bubbles actuated by acoustic forces with a zigzag microstructure. Under proper operating conditions, they achieved a mixing efficiency as high as 0.95 for highly viscous glycerol within a time less than 1 s. Kaba et al. [16] for the first time employed the cavitation microstreaming effect to enhance chemical lysis and DNA extraction in a microfluidic channel. This method overcomes the limit of ineffective mass transport due to a low Reynolds number, and exhibits excellent performance compared to the traditional methods. The aforementioned methods using acoustically actuated oscillatory bubbles, although effective, usually suffer from the inconvenient bubble-trapping process and unstable bubble structure during operation [17]. In addition, it is a complex and far from easy task to generate necessary bubbles directly inside a microfluidic channel due to the challenges in energy concentration, viscous surface force and low number of cavitation nuclei [13,17]. 

Recently, we reported a novel design of a microfluidic channel with PZT actuations for the direct generation of bubbles [18]. The dynamics of a single bubble under actuations was investigated and the mechanism of bubble translation was discussed by studying the interaction force between an oscillating bubble and a boundary wall. In the present study, we investigate the dynamics of a group of cavitation bubbles, which are more frequently observed in the microfluidic channel with actuations. The dynamic behaviors of bubbles under actuations, including oscillation, coalescence and breakup, etc., are recorded and analyzed using high-speed photography. Numerical simulation is also carried out using the commercial CFD software Ansys Fluent, and the pressure field in the microfluidic channel with and without actuations is investigated.

## 2. Materials and Methods

### 2.1. Experimental Setup

**Figure 1:**
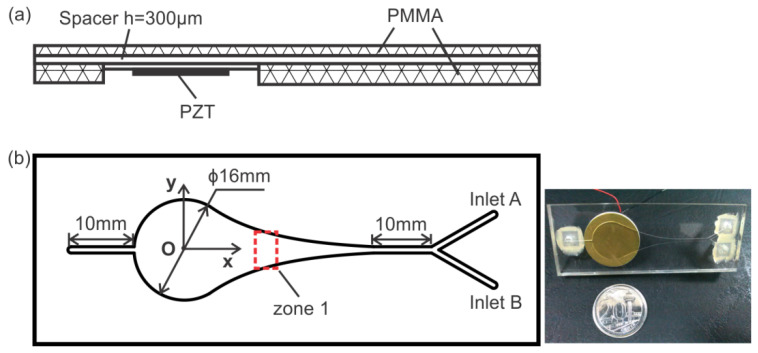
Schematic illustration of the microfluidic channel. (**a**) Side view. (**b**) Top view. The rectangular region zone 1 is selected to record the dynamic behavior of bubbles.

As shown in Figure 1, the microfluidic channel contained two parts—a circular chamber of 16 mm in diameter and a nozzle with an acoustic resonator profile to achieve high-amplitude pressure fluctuations. The microfluidic channel was constructed from two PMMA (polymethylmethacrylate) plates sandwiching a dry adhesive layer 300 µm in thickness (Arclad 8102 transfer adhesive, Adhesives Research, Inc., Glen Rock, PA, USA). Two inlets were connected at a 60-degree angle to the circular chamber by a straight channel 10 mm in length and 1 mm in width, and another straight channel was connected to the other end of the chamber to form the outlet. A PZT disk was attached to the bottom of the circular chamber to provide actuation. The PZT disk consisted of a piezoelectric ceramic layer 15 mm in diameter and a brass sheet 22 mm in diameter. The height of the channel was controlled by the thickness of the adhesive layer. The geometry of the entire configuration was precisely manufactured by a laser cutting machine (Universal M-300 Laser Platform, Universal Laser Systems Inc., Arizona, AZ, USA). Finally, the inlets and outlet were attached to the plate using epoxy glue (Araldite, Huntsman Advanced Materials, Los Angeles, CA, USA).

**Figure 2:**
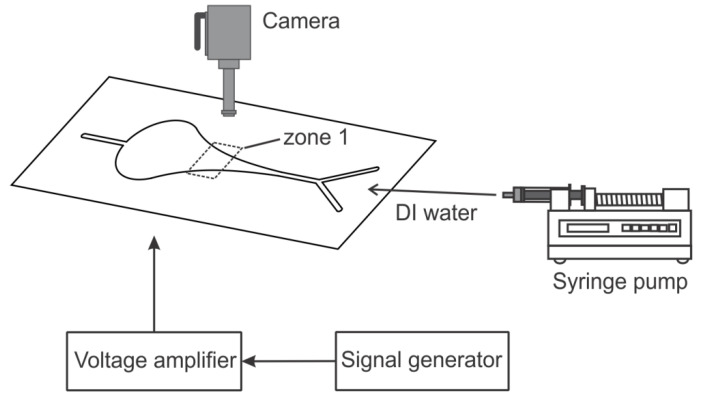
Schematic illustration of the experimental setup.

The schematic of the experimental setup is illustrated in Figure 2. The working fluid, deionized (DI) water, was supplied to the microfluidic channel through two inlets. The flow rate was controlled by two syringe pumps (KD Scientific Inc., Holliston, MA, USA). The piezoelectric disk was driven by an external signal generator (33120A, Hewlett Packard, Palo Alto, CA, USA) and an amplifier (790, PCB Piezotronics, Depew, NY, USA). A high-speed CCD camera (Phantom V711, Vision Research, Wayne, NJ, USA) was mounted on top of the chamber to record the flow field and bubble motion. In the experiments, we focused on the motion of the generated bubbles moving through zone 1 (indicated in Figure 2). The actuation frequency was fixed at 1 kHz and the applied voltage was varied within the range of 120 to 150 V. The flow rate was 10 mL/h in total.

### 2.2. Numerical Simulation

In order to explore the mechanism of bubble generation and oscillation in the microfluidic channel under actuations, three-dimensional numerical simulation was conducted by using the commercial software Ansys Fluent. 

**Figure 3:**
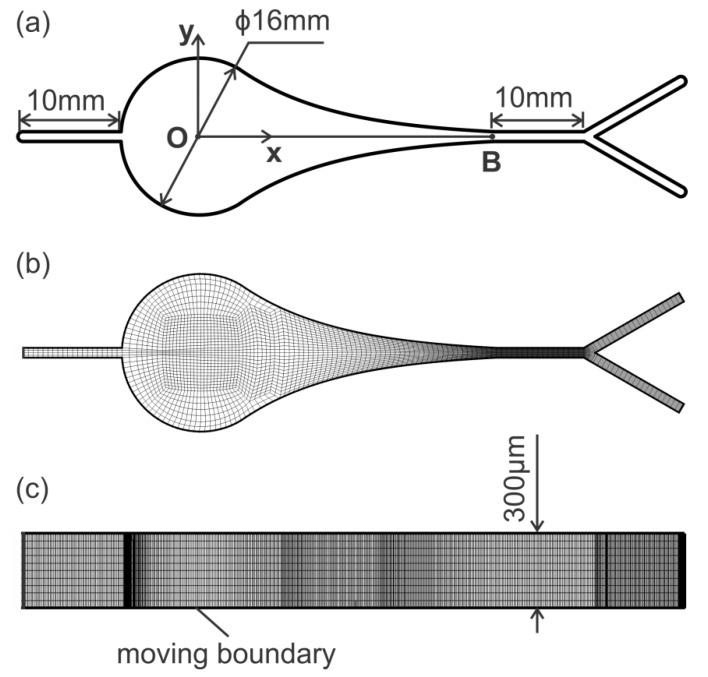
(**a**) Geometry and dimension of the numerical simulation domain. (**b**) Meshing scheme in x–y plane (not to scale). (**c**) Meshing scheme in the height direction (not to scale).

The bubbles generated inside the channel are associated with cavitation of DI water under low-frequency actuations. Thus, the pressure variation and distribution are key issues to understand the mechanisms of bubble generation in the microfluidic chamber. Due to small dimensions of the microfluidic channel, it is difficult to measure the pressure experimentally. Here, numerical simulations using Ansys Fluent were performed to study the pressure variation and distribution in the chamber. The configuration and dimensions of the microfluidic channel in the numerical simulation were identical to the experimental device. The computational domain and meshing scheme are shown in Figure 3. There were tens of layers of meshes along the channel height direction and the meshes inside the chamber and at the Y junction were specifically refined. 

Under the assumptions of an incompressible Newtonian fluid, the governing equations of continuity and momentum are expressed as,

(1) $$\nabla \cdot \vec{V}=0,$$

(2) $$\frac{\partial \left(\rho \vec{V}\right)}{\partial t}+\nabla \cdot \left(\rho \vec{V}\vec{V}\right)=-\nabla p+\mu \nabla^{2}\vec{V}$$

where $\vec{V}$ is the velocity vector, *p* the pressure, *ρ* the density and *μ* the dynamic viscosity.

In accordance with the experiments, the top wall was stationary and the bottom wall with a PZT disk was set as a moving boundary of the PZT disk. The vibration of the PZT disk is described by the displacement ζ(*x, y, t*) [19],

(3) $$\zeta \left(x,y,t\right)=\{\begin{array}{c} \begin{array}{cc} A_{0}\sin\left(2\pi ft\right){\left[1-{\left(\sqrt{x^{2}+y^{2}}/R_{c}\right)}^{2}\right]}^{2} & \begin{array}{cc} & \mathrm{within}\;\mathrm{PZT}\;\mathrm{disk} \end{array} \end{array}\\ \begin{array}{cc} 0 & \begin{array}{cc} & \mathrm{outside}\;\mathrm{PZT}\;\mathrm{disk} \end{array} \end{array} \end{array}$$

where the amplitude of vibration *A*_0_ is 0.5 μm, the frequency imposed to the PZT disk *f* is 1.0 kHz, and *R_c_* is radius of the circular chamber, which is 8 mm in our experiment.

The boundary conditions for the inlet and outlet were set as the velocity inlet and pressure outlet, respectively. All the boundary conditions are listed in Table 1. To examine the mesh independence, we tested three levels of meshes, based on the same meshing scheme, with a total of 438,648 (coarse), 877,296 (medium), and 1,754,592 (fine) cells. The mesh independence tests showed that the simulation with medium grids provided a good trade-off between accuracy and computational cost [18], and it was thus used in the rest of the present study.

The governing equations Equations (1) and (2) were solved by the finite volume method (FVM) in Ansys Fluent and the displacement of the moving boundary described by Equation (3) was fulfilled by the user-defined-function (UDF). The following solution methods and numerical schemes were used in the calculations: PRESTO! for pressure discretion and second order upwind for momentum discretion; PISO scheme for pressure-velocity coupling; and the first order implicit time-dependent solution for unsteady formulations.

## 3. Results and Discussion

### 3.1. Visualization of Bubble Generation by Fluorescent Dyes

In order to visualize the generation and evolution of bubbles, the degassed DI water solution was supplied to the microfluidic channel from inlet A (indicated in Figure 1b), and the same DI water solution with fluorescent dye was supplied to inlet B. 

**Figure 4:**
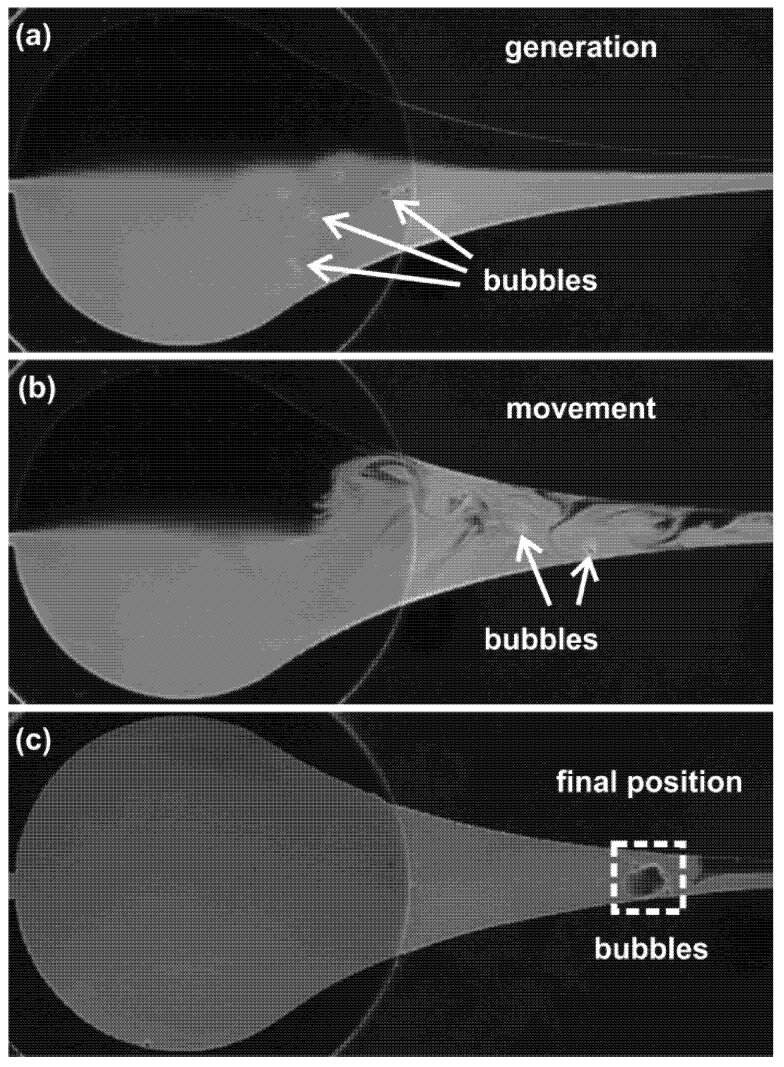
Visualization of the bubbles generated in the channel with actuations at frequency 1.0 kHz and voltage 150 V. (**a**) Bubbles are generated when the actuation is switched on. (**b**) The bubbles travel upstream against the hydrodynamic flow. (**c**) The bubbles arrive at some position close to the nozzle. The flow rate is 10 mL/h in total and the flow direction is from right to left.

The images of the bubbles in the flow at different times are presented in Figure 4. Without the actuation, the upper fluid (without fluorescent dye) and lower fluid (with fluorescent dye) flow separately through the chamber, and no bubbles are observed. As shown in Figure 4a, after the actuation is switched on, a group of small bubbles are found to be generated in the area near the center of the chamber. After being generated, the bubbles travel upstream against the hydrodynamic flow from the inlets (Figure 4b), which results from the bubble–wall interaction. The bubbles finally arrive at some position close to the nozzle and stay in relative equilibrium (Figure 4c). In this process, the violent oscillation, coalescence and breakup of the bubbles cause strong churning of the bulk flow, enhancing the mixing of fluids inside the microfluidic chamber. It was also observed in our experiment that the bubbles can be generated to form as a single bubble, or as a group of bubbles, depending on the applied voltage and the degree of the fluid degassing. Since the dynamics of a single bubble were reported in Ref. [18], we present the study of multiple cavitation bubbles in the next section.

### 3.2. Oscillation and Translation of Bubbles

**Figure 5:**
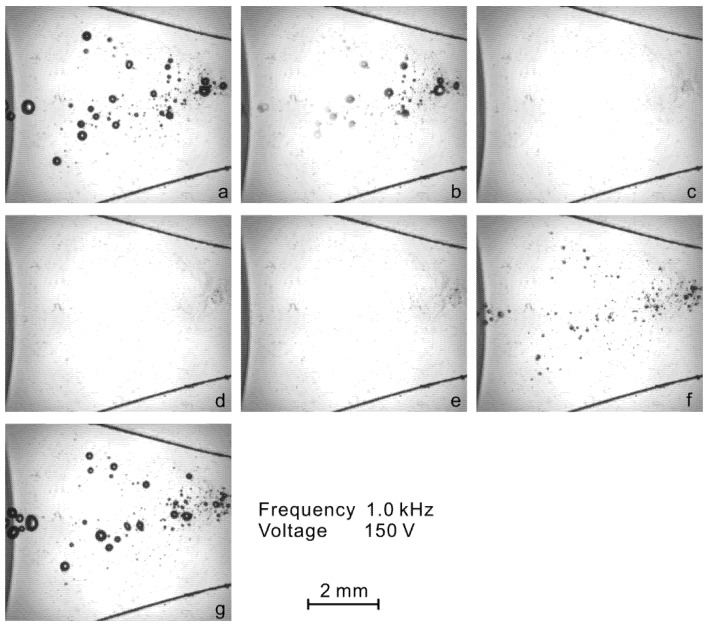
(**a**–**g**) Photographic series of the dynamic behavior of bubbles under actuations in one cycle. The time interval between two successive images is 1/6 T, or 0.167 ms. The actuation voltage and frequency are 150 V and 1 kHz, respectively.

**Figure 6:**
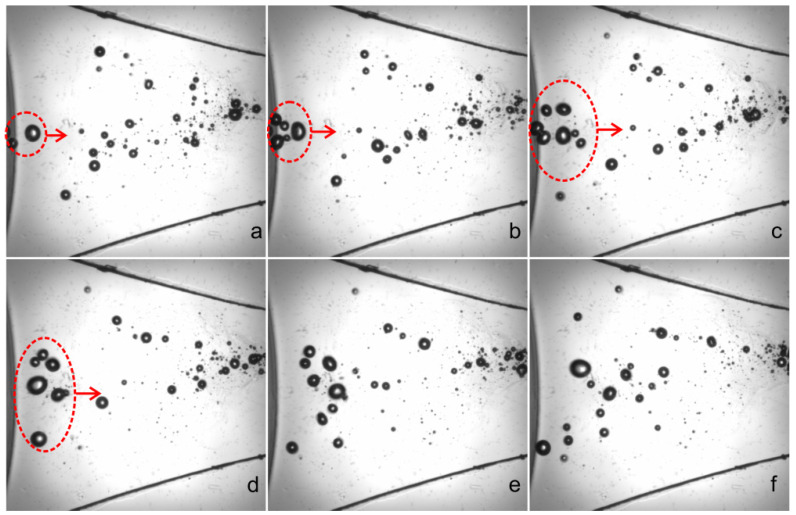
(**a**–**f**) Images of moving bubbles captured at the same phase of different cycles. The time between two successive images is one period—that is, 0.001 s. The actuation voltage and frequency are 150 V and 1 kHz, respectively.

The motions of a group of bubbles moving through zone 1 were recorded by the camera at 12,000 fps, with 12 images captured in per cycle based on the actuation frequency of 1 kHz. The time interval between two consecutive images is one sixth of one period in Figure 5 and Figure 6. It shows that under strong actuations, the bubbles undergo explosive growth and violent collapse alternatively, and during each cycle, the volumes of bubbles are varied by several orders of magnitude: in Figure 5a, the diameter of the largest bubble is 0.45 mm, while in Figure 5c–e, the bubbles are fragmented or even dissolved owing to the compression of actuation. It can also be found that the bubbles undergo drastic coalescence and breakup under the action of actuations. Such a response with the fragmentation or dissolution of bubbles during the collapse phase is termed as “transient cavitation”, which is distinguished from the “stable cavitation” [20]. After their diameters exceed the channel height, the generated bubbles will lose the spherical shape and demonstrate flat surfaces lubricated by thin liquid films near the top and bottom channel walls. 

The time interval between two consecutive images is one period—that is, 0.001 s. At high frequencies, especially in standing waves, bubbles can self-organize into a variety of structures such as streamers, clusters or bubble layers, and evolve on a timescale much larger than the acoustic period. However, in the present study, the bubble clouds are distributed randomly in space without certain structures due to low frequency. For the case of 1 kHz actuation in water, the actuated pressure is almost uniformly distributed in the microfluidic channels, and no acoustic standing waves or travelling waves exist in the microfluidic chamber and thus, the bubbles here cannot be organized into a certain pattern under actuation.

### 3.3. Bubble Coalescence and Breakup

It is known that at low frequencies, the primary Bjerknes force, which results from radiation of the oscillatory pressure field, is very weak, while the secondary Bjerknes force, which is the mutual interaction between oscillating bubbles, is dominant at a short distance. The secondary Bjerknes force can cause the attraction or repulsion of bubbles depending on the phase difference of two bubbles [18]. Therefore, for the bubbles subjected to an oscillatory pressure field, coalescence is further complicated due to the secondary Bjerknes force.

**Figure 7:**
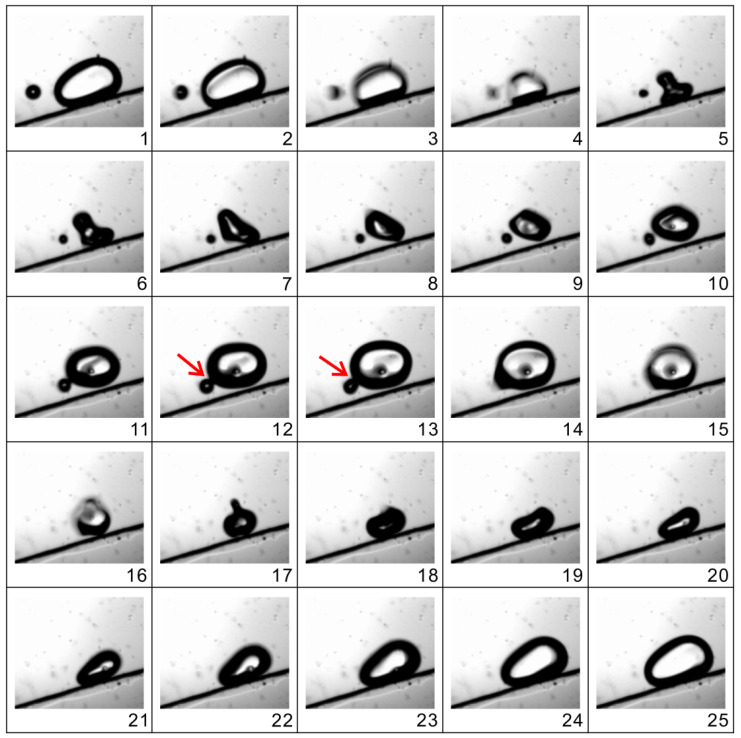
Coalescence of two oscillating bubbles. One small bubble is attracted by a large one and then the two bubbles merge to form a single one. The time between two successive images is 1/12 T, or 0.083 ms. The actuation voltage and frequency are 150 V and 1 kHz, respectively.

The phenomena of coalescence involving two bubbles were photographed and the typical process was exemplified in Figure 7. It is observed that, during the compression phase, a small bubble approaches a large one quickly under the action of the secondary Bjerknes force, and subsequently, at the expansion phase, the two bubbles grow in size until they collide with each other. When the surfaces of two bubbles touch each other, a saddle-shaped “bridge” is built at first and the large bubble immediately “swallows” the small one with the growth of its volume. After the surface area of the resultant cusp decreases, the bubble coalescence is finished. Finally, the new bubble contracts and expands regularly with periodic actuations. The whole process of bubble coalescence occurs during an extremely short time, which is about one third of a period (0.33 ms).

Traditional bubble models such as the well-known Rayleigh–Plesset equation usually assume that the oscillating bubble maintains spherical [21]. However, when a bubble is collapsing steeply, the interface between the dense liquid and less dense gas is violently accelerated in the inward direction and thus, the bubble’s spherical shape is distorted due to the Rayleigh–Taylor instability. Under strong actuations, the shape instability can even cause the breakup of an oscillating bubble.

**Figure 8:**
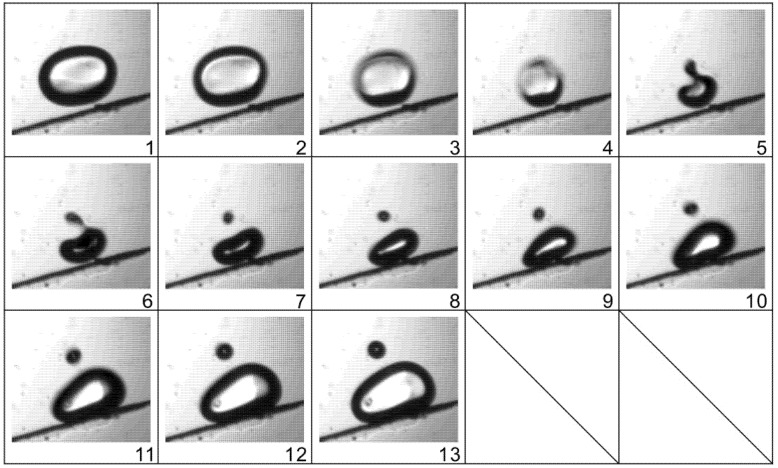
Breakup of one oscillating bubble at the phase of violent collapse. The time between two successive images is 1/12 T, or 0.083 ms. The actuation voltage and frequency are 150 V and 1 kHz, respectively.

An example of bubble breakup is demonstrated in Figure 8. Contrary to the process of coalescence, as the bubble collapses sharply, a cusp is first expelled from the base bubble and then a “neck” connecting the two parts of the bubble is thinned quickly. When the interfacial tension is no longer able to keep the bubble intact, a small bubble is shed from the main one. The whole process of bubble breakup is even faster than coalescence, and takes only one fourth of a period, namely 0.25 ms. Two mechanisms for bubble breakup in fluids have been reported—the inertial mechanism, when the shear stress at the interface overcomes the surface tension, and the resonance mechanism, when the exciting frequency matches the natural frequency. The natural frequency of a micron-sized bubble is higher than 10 kHz, far beyond the applied actuation frequency (1 kHz), and thus, here the bubble breakup is ascribed to the great shear stress caused by large velocity gradients on the two sides of the interface.

**Figure 9:**
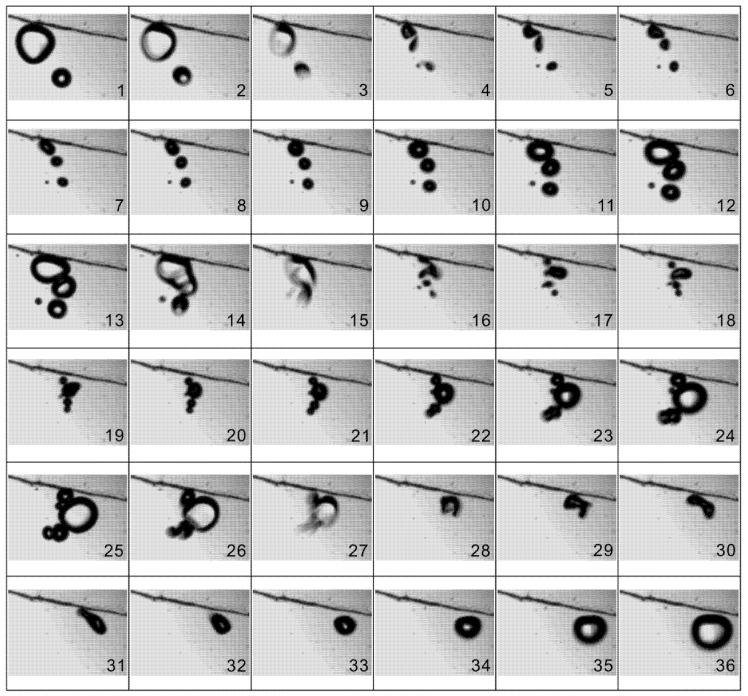
An example of bubbles undergoing alternative coalescence and breakup in the process of translation. The time between two successive images is 1/12 T, or 0.083 ms. The actuation voltage and frequency are 150 V and 1 kHz, respectively.

When bubbles are translated forward, coalescences and breakups happen frequently. Figure 9 shows an example of two bubbles undergoing a series of breakup and coalescence processes in a very short timeframe (three cycles). At first, one bubble breaks up into two smaller ones during the collapse phase and immediately the two resultant bubbles merge into another large bubble, and during the collapse phase of the next cycle, the new large bubble ruptures again into several satellite ones. As a result, after alternating breakup and coalescence over several cycles, the bubble clouds merge into a single bubble. The whole process of successive breakup and coalescence of the bubbles causes vigorous churning of the surrounding liquid, which plays a critical role in mixing the dyed fluids, as shown in Figure 4.

### 3.4. Bubble Generation by Low Pressure

**Figure 10:**
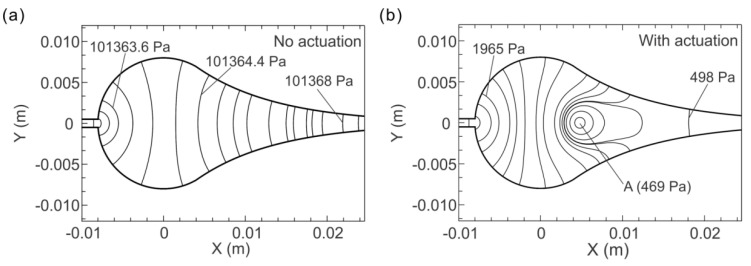
The pressure distributions inside the microfluidic channel by numerical simulation. (**a**) Pressure distribution without actuation. (**b**) Pressure distribution under actuation with frequency *f* = 1.0 kHz at 0.75 T (T = 1/*f* = 0.001 s). The pressure at Point A is 469 Pa.

The simulation results are plotted in Figure 10, which shows the pressure distributions with and without actuations. It can be seen that without actuations (Figure 10a), the DI water flows through the fluidic chamber from right to left with a constant flow rate (10 mL/h). The pressure exhibits a monotonic decrease from the inlet to outlet, and finally is equal to ambient pressure (101,325 Pa) at the outlet, and the pressure drop is slight in the chamber. When the actuation is switched on, the pressure in the channel fluctuates periodically following the actuation. The minimum pressure, which is our focus, in the channel occurs at 0.75 T (T = 1/*f* = 0.001 s) of each period. A typical pressure distribution at 0.75 T is presented in Figure 10b. It is found that, instead of decreasing monotonously, a low-pressure zone occurs in the region of the circular chamber where we observed the bubble generation in the experiments. The minimum pressure is as low as 469 Pa, which is critical to the bubble generation.

Cavitation refers to a process of nucleation from the liquid to the gas phase when pressure falls below some critical value at almost a constant temperature. In real liquid, lots of nuclei or microbubbles exist inevitably, freely suspended or attached to solid boundaries or motes. Those nuclei act at the starting point of cavitation and significantly limit the tensile strength of liquids. It is known that excess pressure is imposed on a microbubble’s surface due to the existence of surface tension, under which a nucleus tends to remain inactive and stable. Thus, cavitation cannot occur unless the applied actuation counteracts the surface tension effect. Considering an oscillating pressure field with the amplitude of *P_a_*, the pressure imposed on the bubble is equal to $P_{0}+P_{a}\sin\left(\omega t\right)$, where *P*_0_ is the static pressure. A microbubble will undergo very weak oscillation and tiny volume pulsation unless the amplitude of the applied pressure fluctuation, *P_a_*, is above a threshold, which is called the Blake threshold [22],

(4) $$P_{a}^{B}=P_{0}-P_{v}+P_{0}\sqrt{\frac{4}{27}\frac{\alpha_{S}^{3}}{1+\alpha_{S}}}$$

where *α_S_* = 2*σ*/(*P*_0_*R*_0_) is the Laplace tension of the bubble in ambient conditions, *P*_0_ = 101,325 Pa and *P_v_* = 3169 Pa are the ambient pressure and vapor pressure, respectively, *σ* = 0.072 N/m is the surface tension and *R*_0_ is the initial radius of the bubble.

Once the amplitude of the pressure fluctuation is higher than the Blake threshold, the minimum pressure is lowered to the critical value, so that the surface tension pressure cannot maintain the bubble’s equilibrium. As such, microbubbles under actuations may expand to a multiple of the initial size and collapse steeply afterwards, undergoing a considerable volume pulsation—bubbles are observed to be “generated” in liquid. The microbubbles in real liquid have characteristic sizes ranging from several microns to tens of microns [22]. Taking a typical microbubble 10 µm in radius (R_0_ = 10 µm), the Blake threshold $P_{a}^{B}$ can be calculated to be 100,111 Pa, corresponding to the threshold pressure on the bubble $P_{L}=P_{0}-P_{a}^{B}$, equal to 1214 Pa. The minimum pressure (469 Pa) from the simulation in the low-pressure zone is significantly below this threshold pressure, and thus the nuclei with radii larger than 10 µm can be “generated” in our experiments.

### 3.5. Volume Pulsation by Pressure Fluctuation

**Figure 11:**
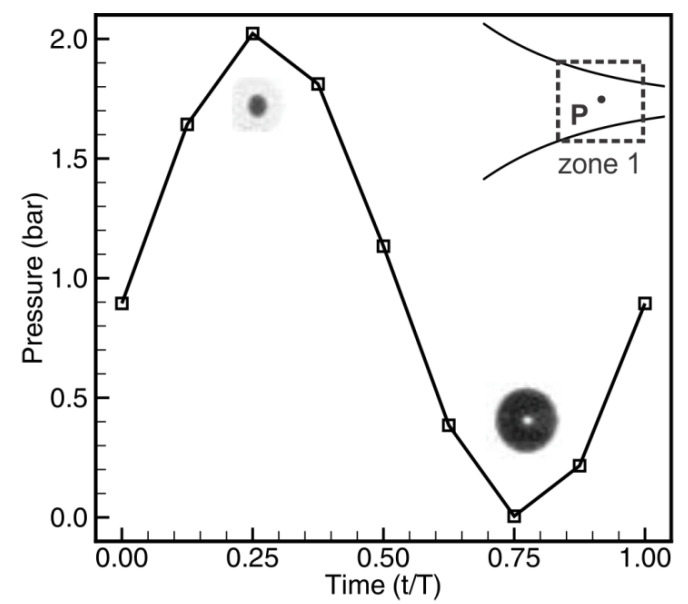
Pressure fluctuation in one cycle at point P (0.013, 0, −0.00015) in zone 1. T = 1/*f* = 0.001 s.

The pressure fluctuation over one period at the point P of zone 1 is plotted in Figure 11. It shows that the pressure fluctuates periodically with its amplitude being about 1 bar following a sinusoidal curve. Under this large amplitude pressure fluctuation, the bubble is expanded corresponding to the minimum pressure, while the bubble is squeezed to a collapse phase at the maximum pressure. These observations can be seen in the inset bubble photos in Figure 11. It should be pointed out that the simulation results are obtained under the conditions of a single phase and incompressible fluid. Once the bubbles are generated in the fluid, these conditions are not applicable and the pressure fluctuation amplitude will be different. However, as the bubble photos show, the actuated pressure fluctuations in the fluid with bubbles have sufficient amplitude to induce a large volume oscillation of the bubbles.

## 4. Conclusions

In this study, the dynamics of cavitation bubbles in a microfluidic channel under actuations are investigated experimentally and numerically. The bubble generation is visualized by tracing fluorescent dyes, and the motions, coalescence and breakup of bubbles are recorded by a high-speed camera. The mechanisms of the bubble dynamics, including generation and oscillation, are studied using numerical simulation.

Bubbles are observed to occur near the center of a chamber under actuations. After being generated, the bubbles translate upstream to a position in a nozzle and simultaneously undergo explosive growth and violent collapse. In this process, the frequent occurrence of the coalescence and breakup of bubbles results in the mixing enhancement of surrounding fluids. Representative examples of bubble coalescence and breakup are provided in detail. The coalescence of oscillating bubbles may be ascribed to the secondary Bjerknes force, and the breakup of an oscillating bubble is caused by the shape instability at the collapse phase. Numerical simulation has been conducted to investigate the pressure variation and distribution inside the microfluidic channel. Simulation results show that during each actuation cycle, there is a low-pressure zone which is exactly the area in which bubble generation is observed in the experiment. The minimum pressure in this zone is found to exceed the Blake threshold corresponding to the hydrodynamic cavitation of pre-existing nuclei, leading to the phenomena of bubble generation in the experiment.

This novel method of bubble generation has the potential to be widely applied for the enhancement of heat and mass transfer in microfluidic channels.

## Figures and Tables

**Table 1:** Boundary conditions used in the numerical simulation.

Component	Boundary Condition	Description
Inlet	Velocity inlet	0.00926 m/s
Outlet	Pressure outlet	Reference pressure: 101,325 Pa
Fluid		Density *ρ* = 998.2 kg/m^3^ Viscosity *µ* = 0.001003 Pa·s
Wall (Bottom)	Moving	
Wall (Others)	Stationary	

## Data Availability

All processed data in this study are included in this published article. Raw data will be provided on request from the corresponding author.
